# PERSISTENT THEMES IN WHERE WE LIVE AS WE ARE LIVING OUR VERY OLD LIVES

**DOI:** 10.1093/geroni/igz038.815

**Published:** 2019-11-08

**Authors:** Miriam S Moss, Sidney Z Moss

**Affiliations:** 1 Arcadia University, Glenside, Pennsylvania, Glenside, Pennsylvania, United States; 2 Arcadia University, Glenside, PA, Northampton, Massachusetts, United States

## Abstract

After living in our suburban house in Pennsylvania for over five decades, as long term gerontologists we moved to Massachusetts. First, we rented a house for three years; then the homeowner decided to sell and we declined to buy. Now in our 90’s, we have been residents for four years of a town house in a retirement community in Massachusetts that emphasizes independence over dependency; it provides no meals or direct health care services. It is now our home and we have found that several themes persevere: (1) We adapt to the increasing presence of vulnerability in ourselves and in other older persons. (2) We try to maximize our sense of residential comfort and mastery (Golant, 2015) by asserting environmental proactivity ( Lawton , 1987). This reflects the continuity (Atchley, 1989) of our lives. (3) We recognize the ever-present complexities in our responses to personal and societal themes of ageism.

